# The Prognostic Value of Serum HBV-RNA during Hepatitis B Virus Infection is Related to Acute-on-Chronic Liver Failure

**DOI:** 10.1155/2022/8422242

**Published:** 2022-09-13

**Authors:** Keli Qian, Ying Xue, Hang Sun, Ting Lu, Yixuan Wang, Xiaofeng Shi

**Affiliations:** Key Laboratory of Molecular Biology for Infectious Diseases, Ministry of Education, Institute for Virus Hepatitis and Department of Infectious Diseases, The Second Affiliated Hospital of Chongqing Medical University, 76 Linjiang Road, Yuzhong, Chongqing 400010, China

## Abstract

**Background:**

Serum HBV-RNA levels can predict antiviral response in chronic hepatitis B (CHB) patients; however, its role in HBV-related ACLF (HBV-ACLF) remains unclear. Here, we determined its implications for HBV-ACLF.

**Methods:**

Baseline serum HBV-RNA levels were retrospectively detected in HBV-ACLF and CHB patients. The association of serum HBV-RNA level with clinical outcomes was evaluated by performing multiple logistic regression. A nomogram was developed to formulate an algorithm incorporating serum HBV-RNA for predicting the survival of HBV-ACLF patients. After being discharged from the hospital, the HBV-ACLF patients were followed up for 36 weeks.

**Results:**

In this study, 82 HBV-ACLF patients and 33 CHB patients were included. Serum HBV-RNA levels were significantly higher in CHB patients than in HBV-ACLF patients (4.15 ± 2.63 log10 copies/mL VS 5.37 ± 2.02 log10 copies/mL) (*P* < 0.05). Among the HBV-ACLF cases, patients with poor outcomes had lower serum HBV-RNA levels, but the difference was not significant. The area under the receiver operating characteristic curve of the serum HBV-RNA inclusive model was 0.745, superior to 0.66 from MELD scores (*P* < 0.05). During the follow-up for four weeks, the serum HBV-RNA levels, especially in the survival group, were found to be lower than the baseline levels.

**Conclusions:**

Serum HBV-RNA levels were associated with disease severity and might predict the long-term clinical outcome of HBV-ACLF patients.

## 1. Introduction

Acute-on-chronic liver failure (ACLF) is a life-threatening syndrome in humans with possible causes including alcohol, drugs, viral infections, and autoimmune hepatitis (AIH) [1, 2]. During decompensated cirrhosis, ACLF displays the characteristics of organ failure with a high rate of short-term mortality [2]. The incidence of ACLF is predicted in one out of four inpatients who experience decompensated cirrhosis, with mortality rates of up to 25% and 40% at 28 and 90 days, respectively [3, 4]. Accurately predicting ACLF can have important clinical effects. By identifying high-risk patients with 28-day mortality, early management of the precipitating factor becomes feasible. This helps to prioritize emergency liver transplantation and plan artificial liver support systems. To help the process, many prognosis prediction systems have also been developed, such as the Child-Pugh score, the Sequential Organ Failure Assessment (SOFA), and the model for end-stage liver disease (MELD) [5–7]. However, these scoring systems are based on the populations of Western countries. Due to regional differences in etiology, alcohol and drugs are the most common causes of ACLF in Western countries, while in China, HBV-related ACLF (HBV-ALCF) accounts for 96.5% of ACLF cases [8, 9].

Completely preventing HBV infections is not possible due to the presence of persistent covalently closed circular DNA (cccDNA) in the affected hepatocyte nuclei [10]. Actively replicating HBV-DNA accounts for an important pathogenic mechanism that initiates and aggravates HBV-related ACLF [11]. Regardless of the status of the HBVe system, the higher levels of HBV-DNA might aggravate the HBV-ACLF, which can be deadly in some cases [12]. Additionally, the administration of lamivudine (LAM) for treatment enhances the long-term prognostic outcomes and reduces the short-term mortality rates among HBV-ACLF cases [13]. The presence of HBV-RNA in serum is considered to be a novel marker of cccDNA [14]. Wang et al. found that HBV-RNA in serum has a stronger relationship with cccDNA compared to HBV-DNA, HBcrAg, or HBsAg, irrespective of the therapeutic outcome [15]. During a chronic HBV infection, the levels of HBV-RNA also change in the serum [16]. However, information on the HBV-RNA levels in the serum of HBV-ACLF patients is limited. Additionally, whether the HBV-RNA content in serum can predict patient prognosis is not known. Thus, in this study, we assessed whether the HBV-RNA content in serum can predict HBV-ACLF.

## 2. Patients and Methods

### 2.1. Patients

The participants included naive chronic hepatitis B (CHB) patients (*n* = 33) and HBV-ACLF patients (*n* = 82) admitted to the Second Affiliated Hospital of Chongqing Medical University between May 2020 and January 2021. We diagnosed the HBV-ACLF patients following the guidelines of the Asian Pacific Association for the Study of the Liver (APASL) [17], while the naïve CHB patients were diagnosed following the Chinese guidelines for the prevention and treatment of CHB (2019 version) [18]. The patients had negative antibodies for antihepatitis A/C/D/E viruses (HAV/HCV/HDV/HEV) and human immunodeficiency virus (HIV). Also, we eliminated cases diagnosed with hepatocellular carcinomas (HCC)/metastatic hepatoma, or autoimmune liver disease. The clinical, viral, and biochemical data were collected at the time of enrollment.

The HBV-ACLF patients were followed up for nine or more months through telephone calls. Also, blood samples were collected in the fourth week in case the patient was still hospitalized. We classified HBV-ACLF cases into death or survival groups based on whether they survived three months after admission. All HBV-ACLF patients were administered tenofovir (TDF), entecavir (ETV), or propofol Tenofovir fumarate (TAF) tablets for antiviral treatment.

The identity of the participants was not revealed. The informed consent of all subjects and/or their legal guardians was obtained for this study. According to the Declaration of Helsinki, the protocol was approved by the Ethics Committee of the Second Affiliated Hospital of Chongqing Medical University.

### 2.2. Laboratory Measurements

We determined the MELD score using the following formula: 3.8 × Ln (TBiL (mg/dL)) + 9.6 × Ln (creatinine (mg/dL)) + 11.2 × Ln (INR) + 6.43 [8]. An Abbott ARCHITECT assay (Abbott, Abbott Park, Illinois, USA) was performed to determine the HBsAg content. The lower limit of detection (LOD) was <0.05 IU/mL. Also, we used the AxSYM automatic rapid immunoassay system (Abbott Diagnostics) to detect the HBeAg content in the serum. A qPCR assay was performed to measure the content of HBV-DNA in the serum. The lower LOD was 100 IU/mL.

### 2.3. Quantification of the Serum HBV-pgRNA

We used the HBV-RNA Quantitative Detection Kit (Sansure Biotech, sansure003) to determine the serum levels of HBV-RNA. The HBV-RNA was isolated from the serum (200 *μ*L), and the sample was incubated with DNase *I* for 30 min at 37°C to digest the HBV-DNA. Then, the First Strand cDNA Synthesis Kit (Roche, Mannheim, Germany) was used to synthesize cDNA. The 2 × RealStar Power Probe Mixture (Gen Star, Beijing, China) was used to conduct qPCR at a density of 10^2^–10^9^ copies/mL; the lower LOD was 50 copies/mL.

### 2.4. Statistical Analysis

The levels of HBsAg, HBV-RNA, and HBV-DNA in the serum were presented as logarithmic units (log_10_ IU/mL). The normally distributed data were displayed in the form of mean ± SD, while the nonnormally distributed data were expressed as the median (interquartile range, IQR). Also, for data with normal distribution, we conducted one-way ANOVA and Student's *t*-tests, while for data with nonnormal distribution, we conducted Kruskal–Wallis and Mann–Whitney *U*-tests. Pearson's correlation coefficient was evaluated to determine the correlations among variables. The correlation between the parameters and patient prognosis was analyzed by performing a multivariate logistic regression analysis. We performed the Kaplan–Meier (K–M) analysis to determine patient survival. The SPSS 18.0 (SPSS Inc, Chicago, Illinois, USA) software was used for analyzing the data.

## 3. Results

### 3.1. Demographics and Clinical Characteristics at the Baseline Level

**Figure 1:**
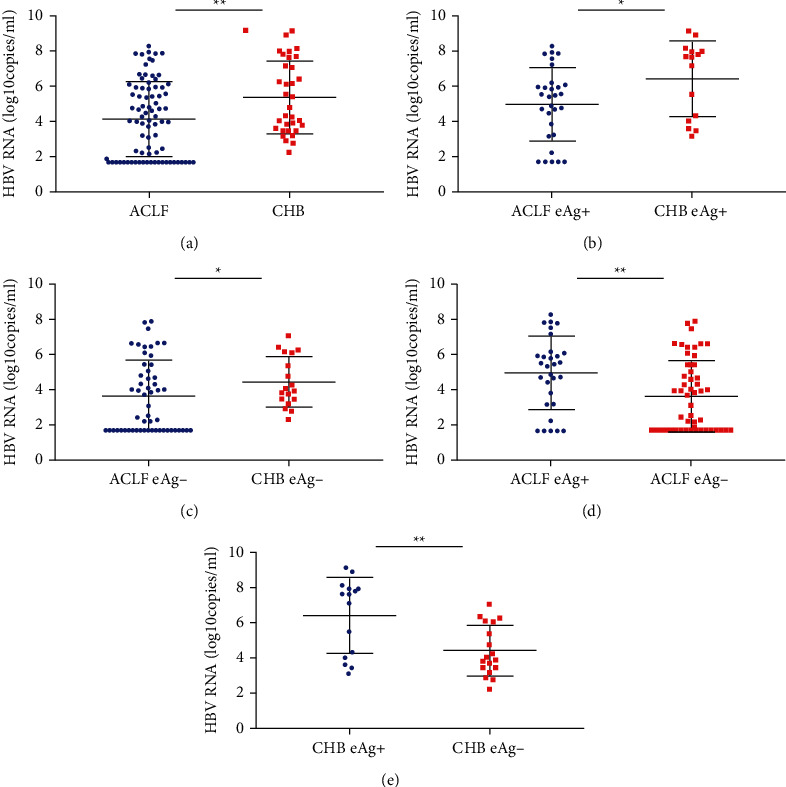
The serum HBV-RNA level in the HBV-ACLF and CHB patients. (a) The mean serum HBV-RNA level (mean ± standard error (SEM)) was significantly lower in the HBV-ACLF patients than in the CHB patients (*P* < 0.01), in HBeAg-positive patients (b) and HBeAg-negative patients (c). In CHB and HBV-ACLF patients, the serum HBV-RNA levels in the HBeAg-positive patients were higher than that in the HBeAg-negative patients (d and e); ^*∗*^*P* < 0.05 and ^*∗∗*^*P* < 0.01.

The characteristics of the CHB and HBV-ACLF patients are presented in Table 1. We included 82 HBV-ACLF patients and 33 CHB patients for further analysis. Patients with HBV-ACLF showed higher ALT, AST, TBil, INR, and lower platelet (Plt) counts than the CHB patients (*P* < 0.05). The HBV-RNA level in the serum of the HBV-ACLF patients was significantly lower than that in the serum of the CHB patients (4.15 ± 2.63 log_10_ copies/mL VS 5.37 ± 2.02 log_10_ copies/mL). Irrespective of the HBeAg status (HBeAg-positive cases: 4.99 ± 2.05 log_10_ copies/mL vs. 6.45 ± 2.09 copies/mL, negative patients: 3.66 ± 2.02 log_10_ copies/mL vs. 4.46 ± 1.41 log_10_ copies/mL), significant differences were found in the HBV-RNA levels (*P* < 0.05) (Table 1 and Figure 1).

Data were expressed as the mean ± standard deviation (SD) for data passed normality check and median (interquartile range, IQR) for data showing a non-normal distribution. ALB, serum albumin; ALT, alanine aminotransferase; AST, aspartate aminotransferase; GFR, glomerular filtration rate; HBV-ACLF, HBV-related acute-on-chronic liver failure; INR, international normalized ratio; Lym, lymphocyte; MELD, model for end-stage liver disease; Ne, neutrophils; PT, platelet; Scr, serum creatinine levels; Tbil, total bilirubin; and WBC, white blood cell.

Among the 82 HBV-ACLF patients, 46 patients survived, with a survival rate of 56.1%, while 36 (43.9%) patients were reported dead after a follow-up of three months. The age of the patients in the survival group was less than that of the patients in the death group. Also, the patients in the death group stayed longer at the hospital (*P* < 0.05). The PT, INR, TBiL, and MELD scores of the survival group were significantly lower than those of the death group (*P* < 0.05). Although the serum HBV-RNA levels of the patients in the survival group were higher than those of the patients in the death group, the difference was not significant (*P* > 0.05). The differences between the groups were not significant for other variables (Table 2).

### 3.2. The Correlation between Serum HBV-RNA Levels and Other Laboratory Parameters

As revealed by Pearson's analysis, the HBV-RNA content in the serum showed a positive correlation with HBV-DNA (*r* = 0.58, *P* < 0.001), HBsAg (*r* = 0.301, *P*=0.006), and HBeAg (*r* = 0.321, *P*=0.003). As for the biochemical indicators, HBV-RNA contents in serum showed a positive relationship with ALT (*r* = 0.325, *P*=0.003), but not with PT, TBil, MELD, or INR scores.

### 3.3. Dynamic Changes in the HBV-RNA Levels of the Serum among the HBV-ACLF Cases

To determine the relationship between HBV-RNA levels in the serum and the length of hospital stay, we analyzed the dynamic changes in the HBV-RNA levels in the serum of 25 HBV-ACLF patients who were still hospitalized in the fourth week. The serum HBV-RNA levels in the fourth week (2.64 ± 1.15 log_10_ copies/mL) were significantly lower than the baseline levels (3.64 ± 2.22 log_10_ copies/mL) (*P*=0.004).

**Figure 2:**
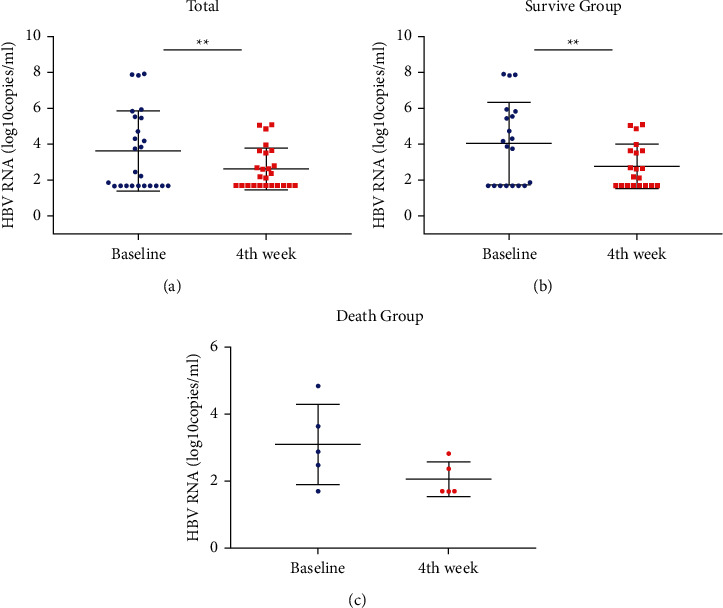
The serum HBV-RNA levels in the HBV-ACLF patients at baseline and on the fourth week. (a) The mean serum HBV-RNA levels (mean ± (SEM)) in 25 patients were significantly lower after four weeks of treatment relative to the baseline levels; the mean serum HBV-RNA levels in the survival group showed a similar pattern (b), but no significant difference was found in the death group (c); ^*∗∗*^*P* < 0.01.

The stratified analysis showed that the serum HBV-RNA levels of the patients in the survival group (*n* = 20) in the fourth week (2.79 ± 1.23 log_10_ copies/mL) were significantly lower than the baseline levels (4.06 ± 2.24 log_10_ copies/mL) (*P*=0.002). The serum HBV-RNA levels were slightly decreased from 3.10 ± 1.19 log_10_ copies/mL to 2.06 ± 0.52 log_10_ copies/mL in the death group (*n* = 5), although the difference was not significant (*P*=0.12) (Figure 2).

### 3.4. Predictive Value of Serum HBV-RNA Levels in the HBV-ACLF Cases

The univariate logistic analysis revealed that neither serum HBV-RNA levels at the baseline nor on the fourth week were related to the survival of the patients. However, the differences in the HBV-RNA levels between the baseline and the fourth week (∆HBV-RNA) were associated with the survival rate (odds ratio (OR) = 0.78; 95% confidence interval (CI): 0.06–0.967; *P*=0.047).

The clinical outcomes showed that for the 25 patients whose serum samples were collected, the area under the characteristic operating curve (AUC) of ∆HBV-RNA was 0.745 (95% CI: 0.52–0.97, *P* < 0.05) while the threshold value was 0.815 log_10_ copies/mL. The specificity and sensitivity were 0.5 and 1, respectively. The MELD score had an AUC value of 0.66 (95% CI: 0.47–0.89, *P* < 0.05) (specificity and sensitivity: 0.53 and 1, respectively).

**Figure 3:**
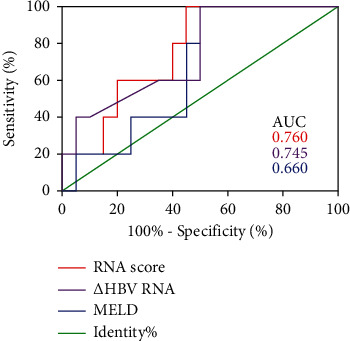
The serum HBV-RNA levels predicted clinical outcomes. The prognostic values of the serum HBV-RNA levels, MELD scores, and RNA scores were assessed by the ROC curves of the HBV-ACLF patients. The numbers represent the corresponding AUC.

To enhance the prediction value, we developed a new prognosis prediction nomogram (RNA score) by introducing the ∆HBV-RNA and MELD scores. The details regarding it are as follows: ∆HBV-RNA-MELD score = −2.70–1.40 × lg [∆HBV-RNA (IU/mL)] + 0.08 × MELD score. The ∆HBV-RNA-MELD score had an AUC value of 0.76 (95% CI: 0.56–0.97, *P* < 0.05), the sensitivity and specificity were 1 and 0.55, respectively, with a cut-off value of 0.17 (Figure 3), but there was no statistical difference among the three prognosis prediction nomograms (*P* > 0.05).

### 3.5. Survival Analysis

**Figure 4:**
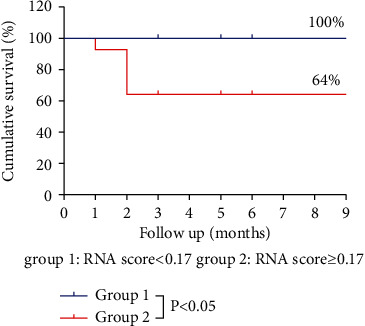
The survival curves were plotted based on the RNA scores used for evaluating the prognosis.

To conduct a survival analysis, all patients were classified into two groups, i.e., group 1 (*n* = 11) and group 2 (*n* = 14), based on the cut-off value of 0.17 (∆HBV RNA-MELD score). The K-M curves, which included the data on a follow-up of nine months, are shown in Figure 4. The results showed that the ∆HBV-RNA-MELD score was strongly associated with mortality due to the differences of the survival parameters between the groups. After a follow-up of nine months, the cumulative survival rates were found to be significantly higher for patients in group 1 (100%; 11/11) than for patients in group 2 (64.29%; 9/14) (*P*=0.03) (Figure 4).

## 4. Discussion

HBV-ACLF is a clinically severe syndrome, and several factors influence its occurrence and progression. In China, chronic infection with HBV is a common cause of ACLF [19]. The activity level of HBV strongly influences the pathogenic mechanism of HBV-ACLF, which is the pathological basis of the disease. Mutations in the HBV basal core or precore promoters can enhance viral replication, which accounts for the main pathogenic mechanism that might induce the progression of HBeAg-negative ACLF [20, 21]. The persistently active cccDNA maintains CHB. Many markers of HBV replication frequently used, including HBV-DNA and HBsAg, cannot accurately predict the intrahepatic activity of the virus [22]. The main strategy for treating HBV infection involves the inhibition of viral replication through the suppression of HBV polymerase. Hence, HBV-DNA has been extensively used for monitoring antiviral treatment [23]. However, nucleotide analogs (NAs) do not influence cccDNA. Therefore, the detection of HBV-DNA has some limitations [24]. Fragments of HBV-DNA are integrated into the human genome to generate serum HbsAg. This suggests that serum HBsAg might not be the best viral marker for cccDNA replication in the liver of CHB patients [25].

The HBV pgRNA in the serum can reflect the transcription of cccDNA in the hepatic tissues of CHB patients [14, 26, 27]. A positive correlation was found between serum HBV pgRNA and most of the commonly used HBV replication markers, such as HBV-DNA and HBsAg by Pearson's analysis in our study. In chronic HBV infection, the average levels of HBV-RNA in the serum differ at different phases; they are higher in HBeAg-positive patients than in HBeAg-negative patients. This pattern was also observed in our study. In both CHB and HBV-ACLF patients, serum HBV-RNA levels were higher in the HBeAg-positive patients than in the HBeAg-negative patients (Figure 1). Chen et al. found that the HBV-RNA levels in the serum were associated with the progression of liver fibrosis [28]. However, studies on the predictive value of serum HBV-RNA levels in HBV-ACLF patients are limited.

This is the first study to assess the value of HBV-RNA levels in the serum of HBV-ACLF patients. The serum HBV-RNA level was significantly correlated with the pathological scores of necrotic inflammation [29]. One study found that the serum HBV-RNA level was substantially higher in patients with elevated ALT levels than in those patients with lower ALT levels [30]. In our study, the serum HBV-RNA levels were positively related to the ALT levels in patients with HBV-ACLF, which is consistent with other studies. But no correlations between PT, TBil, MELD, or INR scores and HBV-RNA levels were observed. We think this is due to the limited sample size. Furthermore, a study with more samples is needed to warrant this observation.

The serum HBV-RNA is derived from the liver nucleus, and the MELD score is closely related to the residual liver function [31, 32]. In addition, a large number of hepatocytes are necrotic in HBV-ACLF patients [33]. According to our results, the serum HBV-RNA levels were significantly lower in the HBV-ACLF patients than in the CHB patients (Figure 1). The death group had lower HBV-RNA levels in the serum and higher MELD scores at the baseline, which indicated that fewer residual hepatocytes were present in the patients of the death group than in the patients of the survival group. After treatment, the serum HBV-RNA levels did not change significantly in the death cases, while the MELD scores increased. This reflected the parenchymal necrosis levels. Also, the number of hepatocytes decreased during the development of HBV-ACLF due to cytokine-related secondary immune injury. We found that the parenchymal necrosis/fibrosis levels were related to the outcome of ACLF, as shown in another study [34]. In the survival group, the MELD scores decreased after treatment, suggesting a decrease in the number of necrotic liver cells. The liver cells regenerated as the liver function improved. However, after Neucleos(t)ide analogues (NAs) antiviral treatment, the serum HBV-RNA levels decreased, which was similar to the findings of Wang et al. [15]. A study by Diogo et al. showed that cccDNA conversion was faster than previously suggested, with a half-life of 5.6 weeks to 21 weeks [35]. Our study supported this finding, considering that the serum HBV-RNA levels of our patients decreased after four weeks of antiviral therapy. However, the mechanism associated with the change needs to be investigated.

Our study showed the value of ∆HBV-RNA in predicting prognosis, and we also established a novel model where the ∆HBV-RNA and MELD scores were used to predict patient prognosis, which can improve the area under the ROC curve. However, our sample size was too small to reflect the statistical differences among the three models.

Our study had some limitations. First, only a few patients participated in the study. Therefore, further studies with more patients needed to be conducted. Also, only Chinese patients with viral genotype B or C were enrolled. We did not compare genotype differences as the relevant data were lacking. More studies need to be conducted that include patients of different genotypes.

Overall, our findings suggest that serum HBV-RNA levels might serve as a prognostic marker for HBV-ACLF.

## Figures and Tables

**Table 1:** The baseline characteristics of the population.

Characteristics	HBV-ACLF *n* = 82	CHB *n* = 33	*P* value
*Demography*			
Male, *n* (%)	65 (79.27)	24 (72.73)	0.57
Age, years	47.18 ± 11.44	43.29 ± 10.28	0.23
Cirrhosis, *n* (%)	29 (35.4)	NA	

*Treatment*			
ETV, *n* (%)	56	NA	
TDF, *n* (%)	24	NA	
TAF, *n* (%)	1		
MELD score	23.19 ± 4.66	NA	

*Laboratory results*			
HBeAg positive, *n* (%)	30 (36.59)	15 (45.45)	0.18
qHBsAg, log10 IU/mL	2.62 ± 1.37	2.98 ± 1.75	0.26
qHBV DNA, log10 IU/mL	5.51 ± 2.04	5.98 ± 1.57	0.71
qHBV RNA, log10 copies/mL	4.15 ± 2.13	5.37 ± 2.02	0.006
INR	2.92 ± 1.41	1.2 ± 0.3	0.001
Alb, g/L	30.91 ± 4.06	41.28 ± 3.78	0.014
TBil, mg/dL	17.01 (4.30, 29.51)	0.84 (0.58, 2.74)	0.001
ALT, IU/L	411.00 (34.28, 2068.17)	48.11 (11.25, 218.69)	0.001
AST, IU/L	352.00 (29.30, 1744.29)	35.26 (7.18, 198.21)	0.001
Scr, mg/dL/L	0.64 (0.47, 1.43)	1.24 (0.97, 1.87)	0.001
WBC, 10^^9^/L	6.64 (4.18, 9.87)	5.84 (3.29, 8.86)	0.28
Ne, 10^^9^/L	4.82 (3.39, 7.07)	5.14 (4.18, 8.29)	0.39
Plt, 10^^9^/L	89.78 (64, 134)	188 (108, 297)	0.001
Lym, 10^^9^/L	1.78 (0.65, 1.39)	1.56 (0.38, 1.76)	0.49

**Table 2:** The comparison of the surviving and nonsurviving patients with HBV-ACLF.

Characteristics	Surviving *n* = 46	Nonsurviving *n* = 36	*P* value
*Demography*			
Male, *n* (%)	36 (78.26)	29 (80.56)	0.47
Age, years	43.93 ± 11.04	51.11 ± 10.57	0.012
Cirrhosis, *n* (%)	13 (28.26)	16 (44.44)	0.123

*Treatment*			
ETV, *n* (%)	12 (26.09)	12 (33.33)	0.22
TDF, *n* (%)	32 (26.57)	24 (66.67)	0.84
TAF, *n* (%)	1 (0.02)	0 (0)	0.79
Hospital stay, days	42 (28, 50)	18 (10, 39)	0.004

*HBV parameters*			
HBeAg^+^, *n* (%)	20 (43.48)	10 (27.78)	0.147
HBsAg, IU/mL	3.10 ± 1.68	2.82 ± 1.67	0.589
HBV DNA, IU/mL	5.76 ± 1.54	5.14 ± 2.48	0.192
qHBV RNA, log10 copies/mL	4.44 ± 1.39	3.90 ± 2.35	0.705
MELD score	21.78 ± 4.69	24.39 ± 3.07	0.007

*Laboratory results*			
PT, *S*	26.69 ± 4.72	29.30 ± 5.47	0.001
INR	2.44 ± 0.55	2.74 ± 0.55	0.001
Alb, g/L	30.79 ± 4.02	31.10 ± 3.74	0.28
TBil, mg/dL	15.02 (4.30, 29.51)	18.69 (6.93, 27.65)	0.001
ALT, U/L	401.28 (34.28, 1923.58)	438.51 (66.72, 2068.17)	0.79
AST, U/L	365.27 (65.28, 1744.29)	313.44 (29.36, 1697.41)	0.47
Scr, mg/dL	0.64 (0.47, 1.43)	0.57 (0.48, 0.99)	0.25
WBC,10^^9^/L	5.17 (4.42, 8.46)	7.92 (5.93, 9.58)	0.38
Ne,10^^9^/L	4.40 (2.89, 6.32)	6.07 (4.18, 7.36)	0.015
Plt,10^^9^/L	95 (74, 137)	89 (60, 132)	0.69
Lym,10^^9^/L	1.06 (0.48, 1.39)	0.81 (0.25, 1.27)	0.001

## Data Availability

All data generated or analyzed during this study are included in this published article.

## References

[1] Granito A., Muratori P., Muratori L. (2021). Acute-on-chronic liver failure: a complex clinical entity in patients with autoimmune hepatitis. *Journal of Hepatology*.

[2] Hernaez R., Solà E., Moreau R., Ginès P. (2017). Acute-on-chronic liver failure: an update. *Gut*.

[3] Hernaez R., Kramer J. R., Liu Y. (2018). Prevalence and short-term mortality of acute-on-chronic liver failure: a national cohort study from the USA. *Journal of Hepatology*.

[4] Moreau R., Jalan R., Gines P. (2013). Acute-on-chronic liver failure is a distinct syndrome that develops in patients with acute decompensation of cirrhosis. *Gastroenterology*.

[5] Malinchoc M. K. P., Kamath P. S., Gordon F. D., Rank J., Rank J., ter Borg P. C. (2000). A model to predict poor survival in patients undergoing transjugular intrahepatic portosystemic shunts. *Hepatology*.

[6] Pugh R. (1973). Transection of the oesophagus for bleeding oesophageal varices. *British Journal of Surgery*.

[7] Ambrosino G., Naso A., Cillo U. (2004). Cytochines and liver failure: modification of TNF-a and IL-6 in patients with acute on chronic liver decompensation treted with molecular adsorbent recycling system (Mars). *Transplantation*.

[8] Gawande A., Gupta G. K., Gupta A., Wanjari S. J., Nijhawan S. (2019). Acute-on-chronic liver failure (ACLF): etiology of chronic and acute precipitating factors and their effect on mortality. *Journal of Clinical and Experimental Hepatology*.

[9] Lan-Lan X., Xiao-Wei K.-Z., Huang Ya-L., Zhao L.-J. (2019). Artificial liver support system improves short-term outcomes of patients with HBV-associated acute-on-chronic liver failure: a propensity score analysis. *BioMed Research International*.

[10] Nassal M. (2015). HBV cccDNA: viral persistence reservoir and key obstacle for a cure of chronic hepatitis B. *Gut*.

[11] Tsai W. L., Lo G. H., Hsu P. I. (2008). Role of genotype and precore/basal core promoter mutations of hepatitis B virus in patients with chronic hepatitis B with acute exacerbation. *Scandinavian Journal of Gastroenterology*.

[12] Lai J., Xie D. Y., Chong T. Y., Yao Z. G., Mo Q. W. (2019). Dynamics of serum HBV DNA levels and meld scores in fatal acute-on-chronic hepatitis B liver failure. *International Journal of Infectious Diseases*.

[13] Xiang-Hui Y., Lang X., Yan Z., Li Z., Xiao-Feng S., Hong R. (2014). Prediction of prognosis to lamivudine in patients with spontaneous reactivation of hepatitis B virus-related acute-on-chronic liver failure: using virologic response at week 4. *European Journal of Internal Medicine*.

[14] Wang J., Shen T., Huang X. (2016). Serum hepatitis B virus RNA is encapsidated pregenome RNA that may be associated with persistence of viral infection and rebound. *Journal of Hepatology*.

[15] Wang X., Chi X., Wu R. (2021). Serum HBV RNA correlated with intrahepatic cccDNA more strongly than other HBV markers during peg-interferon treatment. *Virology Journal*.

[16] Liu Y., Jiang M., Xue J., Yan H., Liang X. (2019). Serum HBV RNA quantification: useful for monitoring natural history of chronic hepatitis B infection. *BMC Gastroenterology*.

[17] Shiv K. S. A. K., John A. A., Chawla Y. K. (2019). Acute-on-chronic liver failure: consensus recommendations of the Asian Pacific association for the study of the liver (APASL): an update. *Hepatology International*.

[18] Chinese Society of InfectiousDiseases CMA (2020). Chinese Society of Hepatology CMA: the guidelines of prevention and treatment for chronic hepatitis B (2019 version). *Journal Prac Hepatology*.

[19] Zhang Q., Li Y., Han T. (2015). Comparison of current diagnostic criteria for acute-on-chronic liver failure. *PLoS One*.

[20] Yang L., Zeng W. T., Liang Z. W., Shi-Wu M. A., Hou J. L. (2013). Basal core promoter/precore and A1846T mutations are associated with hepatitis B-related acute-on-chronic liver failure. *Journal of Tropical Medicine*.

[21] Ren X., Xu Z., Liu Y. (2010). Hepatitis B virus genotype and basal core promoter/precore mutations are associated with hepatitis B-related acute-on-chronic liver failure without pre-existing liver cirrhosis. *Journal of Viral Hepatitis*.

[22] Lesmana C. R. A., Jackson K., Lim S. G. (2014). Clinical significance of hepatitis B virion and SVP productivity: relationships between intrahepatic and serum markers in chronic hepatitis B patients. *United European Gastroenterology Journal*.

[23] Lok A. S. F., Lok A. S. (2013). Hepatitis: long-term therapy of chronic hepatitis B reverses cirrhosis. *Nature Reviews Gastroenterology & Hepatology*.

[24] Ekpanyapong S., Reddy K. R. (2020). Hepatitis B virus reactivation. *Clinics in Liver Disease*.

[25] Saitta C., Tripodi G., Barbera A. (2015). hepatitis B virus (HBV) DNA integration in patients with occult HBV infection and hepatocellular carcinoma. *Liver International*.

[26] Huang Y. W., Takahashi S., Tsuge M. (2014). On-treatment low serum HBV RNA level predicts initial virological response in chronic hepatitis B patients receiving nucleoside analogue therapy. *Antiviral Therapy*.

[27] Giersch K., Allweiss L., Volz T., Dandri M., Lütgehetmann M. (2017). Serum HBV pgRNA as a clinical marker for cccDNA activity. *Journal of Hepatology*.

[28] Liang Chen C. H., Xiaonan Z., Liu S. (2019). Serum HBV-RNA levels is a novel biomarker for liver fibrosis and cirrhosis in chronic HBV infection. *Hepatology International*.

[29] Wang J., Yu Y., Li G. (2018). Relationship between serum HBV-RNA levels and intrahepatic viral as well as histologic activity markers in entecavir-treated patients. *Journal of Hepatology*.

[30] van Campenhout M. J., van Bömmel F., Pfefferkorn M. (2018). Host and viral factors associated with serum hepatitis B virus RNA levels among patients in need for treatment. *Hepatology*.

[31] Botta F., Giannini E., Romagnoli P., Fasoli A., Testa R. (2003). MELD scoring system is useful for predicting prognosis in patients with liver cirrhosis and is correlated with residual liver function: a European study. *Gut*.

[32] Liu S., Zhou B., Valdes J. D., Sun J., Guo H. (2019). Serum hepatitis B virus RNA: a new potential biomarker for chronic hepatitis B virus infection. *Hepatology*.

[33] Bajaj J. S., O’Leary J. G., Lai J. C. (2022). Acute-on-Chronic liver failure clinical guidelines. *American Journal of Gastroenterology*.

[34] Rastogi A., Kumar A., Sakhuja P. (2011). Liver histology as predictor of outcome in patients with acute-on-chronic liver failure (ACLF). *Virchows Archiv*.

[35] Diogo Dias J., Sarica N. (2021). Neuveut C: early steps of hepatitis B life cycle: from capsid nuclear import to cccDNA formation. *Viruses*.

